# Efficacy of Nanosilica Coatings in Calcium Looping Reactors

**DOI:** 10.1021/acs.iecr.2c03490

**Published:** 2023-01-17

**Authors:** F. J. Durán-Olivencia, R. Gannoun, A. T. Pérez, J. M. Valverde

**Affiliations:** † Dpto. de Ingeniería, Universidad Loyola Andalucía, Avda. de Las Universidades s/n, 41704, Seville, Spain; ‡ Facultad de Física, Universidad de Sevilla, Avda. Reina Mercedes s/n, 41012Seville, Spain

## Abstract

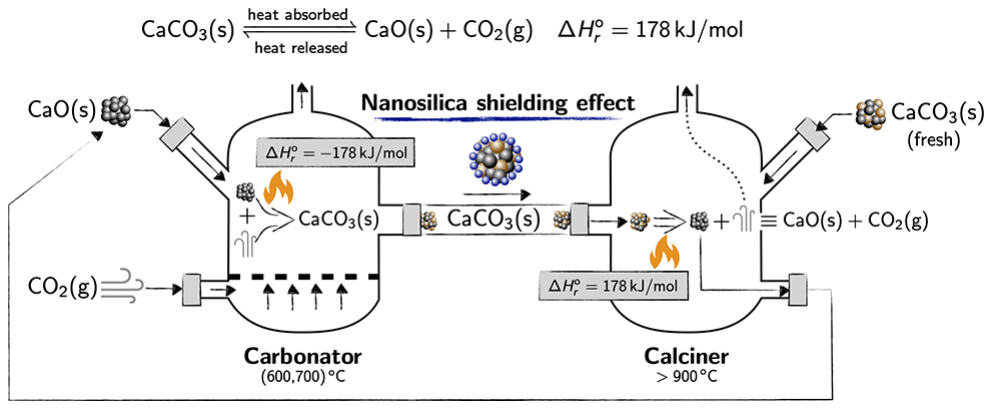

Nanosilica coatings are considered a simple physical treatment to alleviate the effect of cohesion on powder flowability. In limestone powders, these coatings buffer the rise in cohesion at high temperatures. Here, we investigate the role of particle size in the efficiency (and resilience) of these layers. To this end, this work examines a series of four limestone powders with very sharp particle size distributions: average particle size ranged from 15 to 60 μm. All the samples were treated with nanosilica at different concentrations from 0 to 0.82 wt %. Powders were subjected to short- and long-term storage conditions in calcium looping based systems: temperatures that vary from 25 to 500 °C and moderate consolidations (up to 2 kPa). Experiments monitored powder cohesion and its ability to flow by tracking the tensile strength of different samples while fluidized freely. Fluidization profiles were also used to infer variation in packings and the internal friction of the powder bed. Interestingly, for particle sizes below 50 μm, the nanosilica treatment mitigated cohesion significantly—the more nanosilica content, the better the flowability performance. However, at high temperatures, the efficiency of nanosilica coatings declined in 60 μm samples. Scanning electron microscopy images confirmed that only 60 μm samples presented surfaces barely coated after the experiments. In conclusion, nanosilica coatings on limestone are not stable beyond the 50 μm threshold. This is a critical finding for thermochemical systems based on the calcium looping process, since larger particles can still exhibit a significant degree of cohesion at high temperatures.

## 1 Introduction

Cohesion is one of the central issues in granular-based thermochemical storage (TCES) technology.^1^ It affects the flowability of the powder through the storage circuit, making TCES systems especially vulnerable to jammings, interruptions, and eventually shutdowns. This is the primary downside of solid-based solutions to store the solar radiation in concentrated solar (CSP) plants. Yet, granular media represent the major asset for the future of the CSP industry.^2^ Unlike liquid alternatives, granular materials can operate under the extreme conditions that govern the storage line in CSP facilities:^3,4^ a temperature span of 1000 °C.

Certainly, liquids are more efficient media for transporting thermal energy through the storage circuit. However, no conventional material remains liquid in this range of temperatures. For this reason, liquid solutions rely on molten salts shaped by eutectic mixtures of liquid metals. Today, lead–bismuth mixtures, Bi–Pb (LBE), represent one of the most promising candidates in the mainstream.^5^ Although LBE liquids surpass (theoretically) the 600 °C barrier,^6^ their low thermal conductivities and high densities reduce their heat storage capacity. In addition, the integration of these metallic liquids in storage systems poses another critical issue. They exhibit significant corrosion rates^7^ and high solubility limits for nickel and copper. In fact, there is still a lack of fundamental understanding of the materials and protective layers that should be used with these liquids.^8^ Because of these limitations, commercial facilities run by molten salts operate in a quite limited range of temperatures, from 300 to 500 °C.^9^ Otherwise, the liquid could solidify or degrade as the temperature goes beyond those limits.

This is the context that makes granular flows stand out from their liquid counterparts. First, granular flows behave as a dry fluid, which eases the limitation of high temperatures. Second, granular media with small particle sizes offer a massive area to react through grain surfaces. Third, gas–solid reactions allow thermal energy to be stored in the form of chemical potential energy. Thus, reactions with high activation enthalpies result in high energy densities. More importantly, once the reaction takes place in the reactors, byproducts can be separated in different silos. This is the major asset of thermochemical energy storage (TCES) solutions based on granular media. With the reactants separated from each other, storage can be performed at ambient temperature. Therefore, thermochemical storage systems represent a simple but efficient solution that provides short- and long-term energy support with almost zero heat losses.^10,11^

Despite all these conveniences, powders are trickier to optimize. One of the critical points is that granular flows become cohesive at high temperatures. With the rise in cohesion, powder flowability plummets, and the flow regime can quickly shift from a free-flow pattern to an intermittent one with jammings, interruptions, and eventually shutdowns.^12^ Therefore, powder flowability is critical in production environments, which demand smooth and uninterrupted granular flows.

Primarily, two factors modulate the flowability of these powders: particle size^13^ and temperature.^1,14^ As particle size decreases: (1) The ratio between the reactive surface and the particle weight increases; and (2), with smaller particle sizes, cohesive forces dominate over inertial ones, turning the granular flow from inertial to cohesive. Hence, although small particles improve reactivity, they also promote cohesion. Temperature makes the rise in cohesion even more dramatic. In effect, as the temperature approaches the Tamman crossover (the sintering temperature), ions and atoms mobilities in the solid skyrocket, and surfaces tend to soften. As a result, higher temperatures favor wider contact areas, increase the cohesion between grains, bridge the powder, and erode the powder flowability accordingly. This is a critical concern in fluidized bed and entrained-flow reactors,^3,4,15−17^ specially when it comes to the transportation of such cohesive granular flows through the pipelines.

Indeed, previous studies addressed this issue by coupling other phenomena that help to fluidize cohesive granular materials easily. This is the case of acoustic stimuli that hinder the agglomeration in fine particles (<50 μm).^18−20^ However, although sound can modify the flow regime and prevent particles from agglomerating, it cannot avoid the rise in cohesion with temperature. This is because the mechanical stimulation associated with sound does not change the interaction between particles. Therefore, sound does not prevent particles from sintering nor alter their deformation at contact, which triggers the increase in cohesion.

Certainly, this work shares with those studies mentioned above the emphasis on tackling the transportation problem of cohesive granular materials through pipelines. Nevertheless, it leans toward those studies that use nanosilica coatings to contain the deformation of carbonate surfaces at high temperatures. In so doing, this work investigates the efficiency of these coatings under those conditions that characterize thermochemical storage units. Overall, the research targets the flowability issue of transporting cohesive granular media from a microstructural point of view.

So far, nanosilica coatings have proven to be an effective remedy for limestone.^1^ There are clear evidence that show how nanoparticles of silica alleviate the downside of cohesion at high temperatures. Nanosilica easily adheres to carbonates, and the resulting coverage shapes a mechanical shield that offers a higher degree of hardness and thermal resistance than a naked surface of CaCO_3_. The shielding effect of nanosilica layers limits the surface deformation that stimulates cohesion at high temperatures. However, although this is true for limestone particles of around 45 μm, few studies have investigated the role of particle sizes in the efficacy of these coatings.

**Figure 1:**
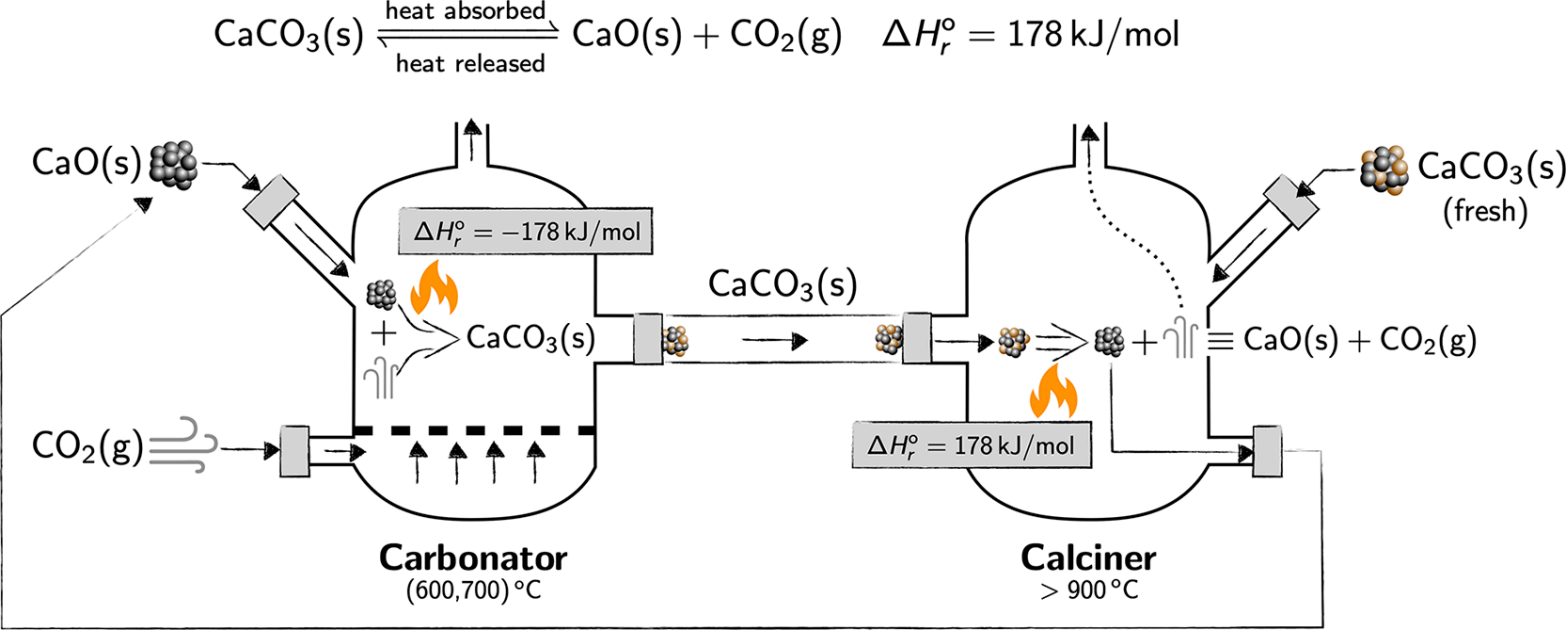
Illustration of the double reactor scheme used in calcium looping (CaL) circuits for concentrated solar plants.

Within this context, this work concentrates on analyzing the efficiency (and resilience) of nanosilica coatings as the limestone particle size increases. To do so, this work extends previous studies about the benefits of nanosilica coatings by analyzing the impact of these layers on different particle size distributions. The results offer, thus, unparalleled feedback about how nanosilica can change the flow regime in production environments, where powders exhibit more often quite scattered particle size distribution. Larger particles, in principle, do not pose a problem when it comes to transportation, as they usually display a lower degree of cohesion. However, this is no longer true at high temperatures, when cohesion increases significantly even in larger particles.^13^ In particular, we focus on exploring one of the most critical sectors in thermochemical storage circuits: the pipelines that transfer carbonates from one reactor (carbonator) to the other (calciner) (Figure 1). In this sector, the use of nanosilica targets the overall performance by two actions: (1) it prevents the use of thermal reductions at the carbonator exit, where carbonates get out at approximately 650 °C; and (2), as the powder is headed to the calciner at higher temperatures, it reduces the thermal leap that carbonates must undergo to activate the reaction in the calciner above 900 °C. Yet, carbonates decompose quickly from 600 °C resulting in calcium oxide and carbon dioxide. Therefore, carbonate transportation from one reactor to the other must occur below this threshold, ensuring that a minimal amount of carbonate degrades before reaching the calciner.

In this scenario, the experiments were conducted monitoring the tensile strength in fluidized beds with four sharp particle size distributions ranging from 15 to 60 μm. First, raw samples were examined at different temperatures (from ambient to 500 °C), thus building a control series to contrast the effect of nanosilica coatings. Subsequently, samples coated with nanosilica were analyzed, varying the nanosilica content from 0 to 0.82% wt. In either case, because packings can be critical in powder dynamics, all the samples were subjected to preconsolidations between 0 and 2 kPa. The results evidence that the efficiency of nanosilica coatings depletes in larger particles (above 50 μm). Scanning electron microscopy (SEM) images back these results, showing surfaces barely coated with nanosilica in 60 μm samples. These outcomes can be valuable for pilot plants, where powders can be treated (generally, sifted, mixed) to optimize the use of nanosilica. The analysis and discussion could also be valuable for devising more efficient nanosilica coatings that may benefit a broader spectrum of particle sizes.

**Figure 2:**
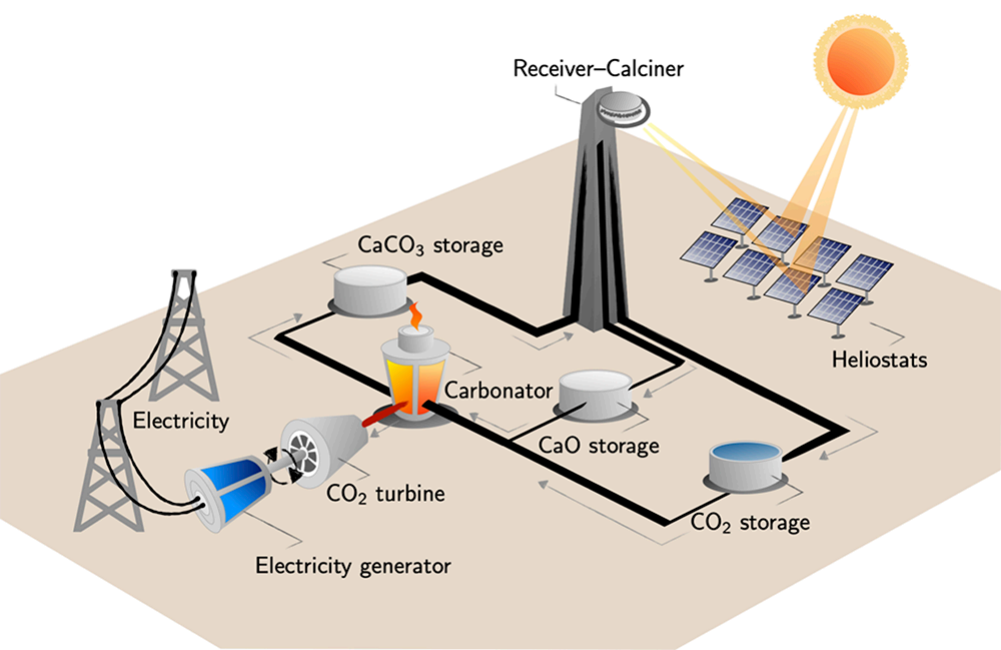
Schematic representation of a concentrated solar power (CSP) plant assisted by a thermochemical unit based on the calcium looping process.

This work has been carried out within the H2020 European project (socratces,^17^Figure 2), coordinated by the University of Seville. The main goal of this project is to demonstrate, on a pilot scale, the suitability of the calcium looping process^21^ for energy storage using fine limestone powders. This technology aims to drive energy storage in new concentrated solar power plants, which demand better mechanisms to control short- and long-term energy storage.

## 2 Materials and Experimental Setup

In what follows, the materials and the experimental setup used in this work are introduced in detail.

### 2.1 Materials

**Figure 3:**
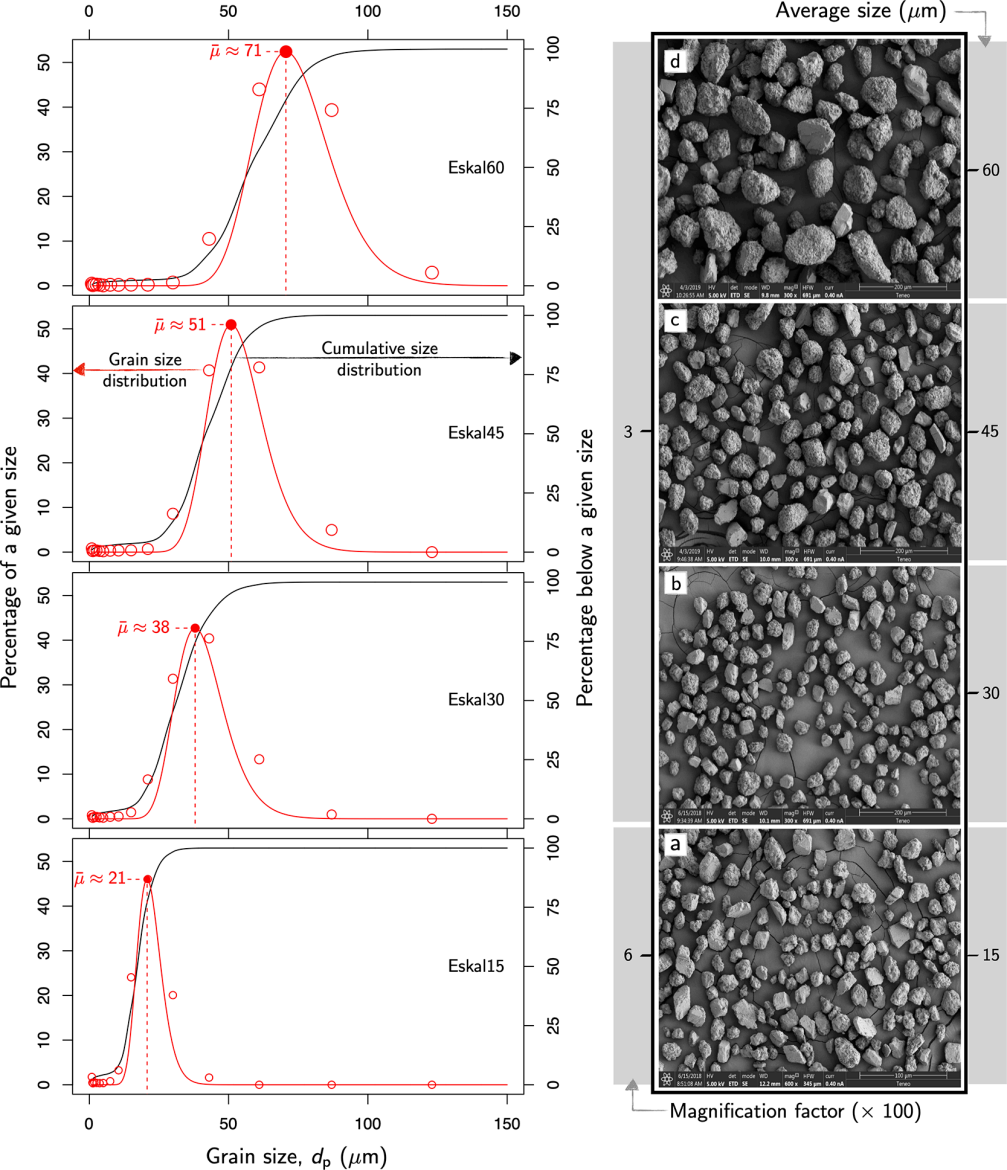
Particle size distribution used in this work for limestone powders (99.1%CaCO_3_, supplied by KSL Staubtechnik Gmb: https://www.ksl-staubtechnik.de. Pictures on the right side show the corresponding scanning electron microscopy (SEM) images.

This work examined a series of four well-differentiated particle size distributions (Figure 3), drawing attention to the efficiency and stability of these coatings as the particle size increases.

Each of the control samples for fine limestone (99.1% calcium carbonate, CaCO_3_, supplied by KSL Staubtechnik Gmb) exhibited a sharp distribution around the average particle size (Figure 3). This critical detail makes it possible to control potential size effects that otherwise would be concealed by the convolution of different sizes.

The experiments were performed on two types of limestone samples: raw and coated with nanosilica (Aerosil R974, from Evonik Industries). Raw powders were used as control samples to analyze the evolution of cohesive forces as the nanosilica content increased to grow thicker layers. To this end, the content of nanosilica was varied from 0 to 0.82% wt. The dry-mixing process, and subsequent coatings, are driven by a rotating drum spinning at 55 rpm that energizes the powders for 1 h.^22^ This technique has proven to be effective in stimulating uniform and stable nanosilica layers in carbonates (CaCO_3_). It relies on the opposite triboelectric character of silicates and carbonates, respectively; these two components tend to be charged with different polarities as they collide with each other. Consequently, as the mixture is stirred, collisions build the electrical attraction that eventually shapes stable and uniform coatings. Further details of this procedure can be found in the literature.^22,23^

Table 1 compiles those properties used in this work to discuss and analyze the evolution of the contact and the powder cohesion: average particle diameter *D*_*p*_, particle density ρ_*p*_, solid mechanical hardness *H*, Young’s modulus *E*, Poisson ratio ν, and surface energy γ.

**Table 1:** Material Properties at Room Temperature Reported in the Literature for the Powders Tested in This Work

Materials	Density ρ_*p*_(kg/m^3^)	Diameter *D*_*p*_ (μm)	Mechanical hardness *H* (GPa)	Young’s modulus *E* (GPa)	Surface energy γ (J/m^2^)	Poisson ratio ν (−)
CaCO_3_	2700^a^	41.52^a^	(25, 88.19)^b^	(0.75,5.11)^c^	(0.21, 0.34)^d^	(0.32, 0.35)^e^
SiO_2_ (fumed silica)	2200^a^	≈0.1^a^	74^f^	6^f^	0.17^g^	0.025^h^

Source.

a Data provided by the supplier.

b References (72−79).

c References (73, 76, 77, 80, 81).

d References (72−75, 82−85).

e References (85, 86).

f Reference (22).

g Reference (87).

h Reference (88).

### 2.2 Experimental Setup

**Figure 4:**
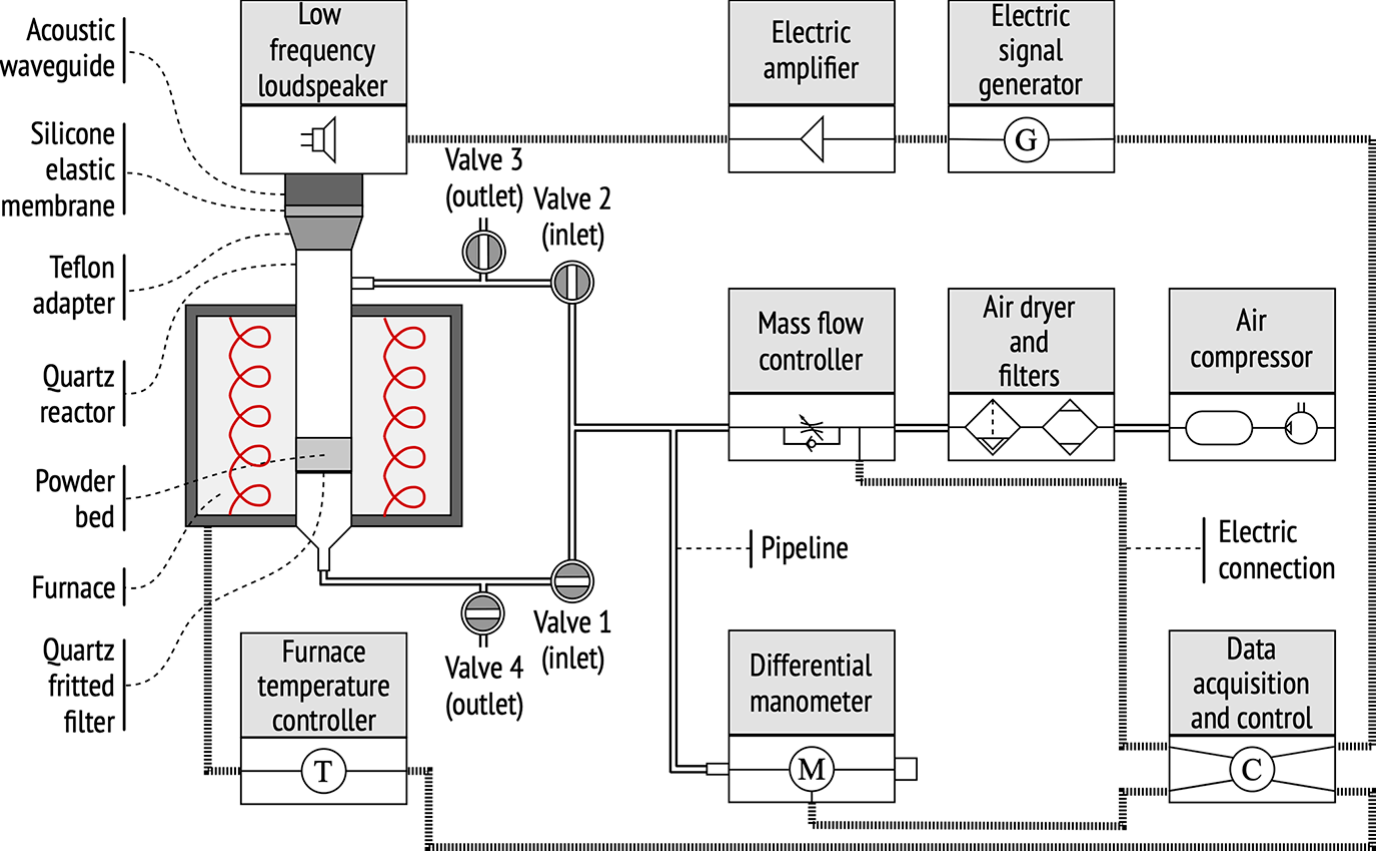
Experimental setup used to measure the tensile strength of powders as a function of temperature. Data acquisition is automated via LabView.^14^

The experiments were performed using a variant of the powder tester proposed by Valverde et al. (Seville Powder Tester — SPT). In the past years, the SPT setup has been extensively used to characterize a wide spectrum of powders.^22,24−29^ This series of experiments was conducted using the last upgrade of SPT (High Temperature Seville Powder Tester — HTSPT, Figure 4). Among the evolutions of this new setup, three of them make it especially suitable for this series of experiments: (1) it can operate at high temperatures^1,14,30^ (up to 1000 °C); (2) it introduces a sound generation system that can be used to stimulate and randomize the initial powder stage; and, (3) it is fully managed by a computer, which reduces the human factor and its potential impact on powder memory effects. Further details about the HTSPT protocol can be found in previous works.^1,14,30,31^

The advantage of the HTSPT technique compared to shear stress testers is that it gauges the tensile strength of the powder by tracking the fluidization curve. HTSPT monitors the pressure drop through the powder bed while this expands and fluidizes freely. Thus, HTSPT facilitates the analysis of those properties and mechanisms that modulate powder fluidization. Cohesion is one of the most important properties to foresee the potential side effect in thermochemical units.

Figure 4 sketches the experimental setup. Samples settle on a porous ceramic plate located at the bottom of a vertical cylindrical quartz tube of 4.5 cm diameter. Although permeable to the gas, this ceramic plate is impermeable to powder particles. An upward airflow is supplied through the porous plate to track and analyze fluidization profiles. As the upstream flow traverses the powder bed, the powder tends to expand and fluidize accordingly. The amount of powder is chosen carefully to keep the height of the bed (about 2.8 cm) below the diameter of the cell. This prevents wall retention effects from intervening in the fluidization regime.^32^

Furthermore, traces of humidity or other pollutants in the airflow could also distort the fluidization dynamic. For this reason, the airflow is filtered and dried before being pumped through the bed. The circuit is equipped with a set of filters and an air dryer (model SMC IDFA3E) that clean the air stream from both moisture and pollutants.

Regarding how the fluidization regime is handled, a couple of mass flow controllers (Omega model FMA-2606 A, 2000 sccm) are responsible for modulating the flow rate. These two controllers operate with a set of electrical valves that enable bidirectional flows through the samples. While upward flows fluidize the bed, downward flows consolidate the sample prior to the fluidization regime. To fluidize the sample (*direct mode*, Figure 4), valves 1 and 3 open, while valves 2 and 4 remain closed. The fluidization curve is logged as the gas flow rate increases at 5 cm^3^/min every 3 s. Before the fluidization stage, samples are consolidated running the circuit in *reverse mode* (Figure 4), in which valves 2 and 4 open, while valves 1 and 2 remain closed. Through this consolidation stage, samples are subjected to downward flows, holding the target consolidation stress for 30 s. While reaching the target stress, the flow rate increases at 5 cm^3^/min every 3 s. In either case, the pressure drop across the bed is monitored using a differential manometer (MKS model 220CD, 10Torr full scale).

The HTSTP setting integrates a sound generation system at the top of the cell. This element is intended to assist powder fluidization and prevent memory effects in the initial state.^33^ Every experiment starts by subjecting the bed to flow rates in the bubbling regime along with an acoustic stimulation of 150 dB at 130 Hz. This combined action prevents memory effects from escalating to any subsequent stage of the experiments. This initialization stage guarantees reproducible results; it takes place after the corresponding thermal stabilization. The target temperature is set for at least 1 h. After this period, the experimental sequence resumes—first going through the initialization stage, later running the consolidation phase, and finally performing the fluidization series.

Further details about the protocol can be found in previous works.^1,14,30,31^

## 3 Results

### 3.1 Fluidization Curves

**Figure 5:**
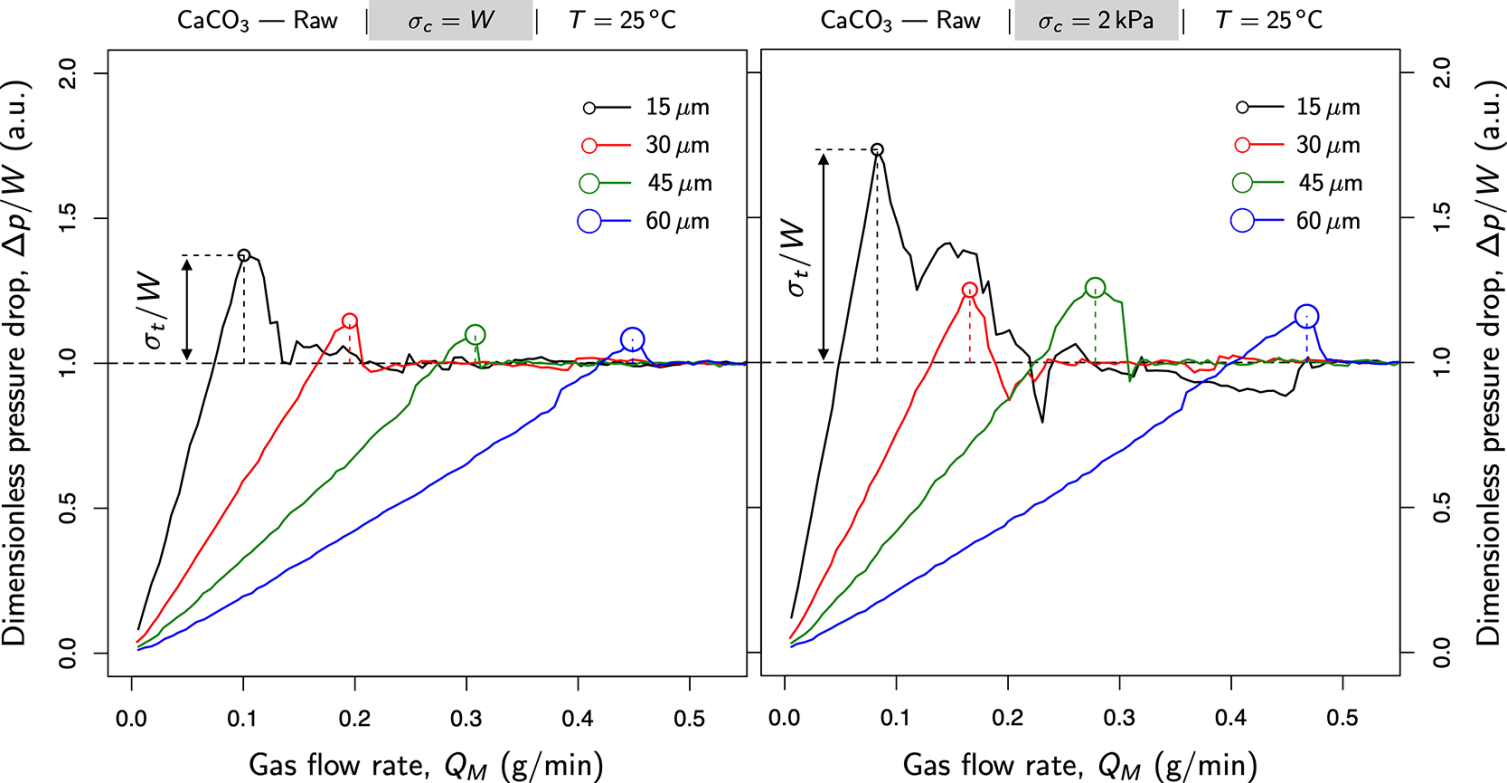
Packing effect under standard laboratory conditions: 25 °C and 1 atm. The gas pressure drop (Δ*p*) across the powder bed is plotted as a function of the mass flow rate; Δ*p*/*W* refers to the pressure drop in arbitrary units using the sectional weight of the powder bed (*W* ≈ 370 Pa).

Figure 5 profiles the fluidization curves for raw samples with different particle sizes under standard laboratory conditions: 25 °C and 1 atm. On the left side of Figure 5, those samples consolidated under their own weight (i.e., no additional flow stress was applied), settling under the influence of gravity. This consolidation stress, by default, represents the ratio of weight to cross-sectional area of the powder bed (the sectional weight of the powder bed, *W* ≈ 370 Pa). On the other hand, on the right side of Figure 5, samples were preconsolidated at 2 kPa, which is equivalent to the experiments on the left side but with a gravitational effect of approximately 5 times Earth’s gravity (5*g*).

Solid lines in Figure 5 monitor the pressure drop through the bed as the flow enters the vessel from the porous plate located at the bottom of the cell. The pressure drop was expressed in arbitrary units using the sectional weight of the powder bed, Δ*p*/*W*. Certainly, as the flow passes through the bed, it induces an upward drag force on the particles. This vertical stress depends directly on the pressure gradient and the particle weight. A black dashed line was drawn in both sides of Figure 5 to facilitate reading such a relationship between pressure and weight (Δ*p*/*W* = 1). It indicates the point at which the upward buoyancy equals the powder weight (usually referred to as either the incipient fluidization point or the minimum fluidization condition).

Three regions can be differentiated in all the profiles in Figure 5. The first sector is delimited by the linear trend before the incipient fluidization point, represented by the point at which the profile *y*-intercepts the black dashed line. Within this region, the aerodynamic drag is still not enough to overcome the downward force of gravity. The second area extends the linear trend to a range of mass flow rates that exceed the minimum fluidization condition (Δ*p*/*W* = 1). This stage ends when the powder fractures at the peak. Finally, the third domain refers to the mass flow rates under which the powder exhibits a liquid-like state: the fluidization regime when the pressure drop oscillates around the sectional weight of the powder bed, *W* (the ratio of the total weight of the powder and the cross-sectional area of the bed).

Below the flow rates that trigger the fracture, the pressure drop increases linearly with the mass flow rate (Carman–Kozeny equation):
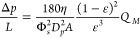
1where Δ*p* refers to the pressure drop through the powder bed, *L* is the total height of the bed, η represents the kinematic viscosity of the gas, Φ_*s*_ describes the sphericity of the particles that made up the granular medium, *A* indicates the cross-sectional area to flow, *D*_*p*_ accounts for the particle size, ϵ means the porosity of the bed, and *Q*_*M*_ alludes to the mass flow rate of the gas.

Nonetheless, eq 1 assumes low Reynolds numbers:^34^
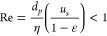
2with *u*_*s*_ representing the superficial gas velocity. According to the data presented in Section 2, eq 2 can be evaluated considering (1) typical porosity values for the powders used in this work, ϵ ≈ 0.5; (2) average particle sizes ranging from 15 to 60 μm; and, (3) superficial velocities below 0.5 cm/s. With this set of values results Re < 0.01, even if temperature is considered in the fluid viscosity.

In light of eq 1, while the flow rate is below the minimum fluidization condition, the powder exhibits a solid-like state. The bed behaves then as a mechanical frame since it can withstand the tensile stress without significant changes in its internal structure. The initial linear stage is, therefore, a byword of a static internal state. Consequently, porosity is expected to remain constant throughout this region, as the drag force is still not enough to overcome the internal friction.

Afterward, when the fluidization curve intercepts the black dashed line, upward and downward forces balance. From this point on, any further increase in the mass flow rate propels and expands the powder bed. However, the linear stage still holds for a while. This is the hallmark of cohesive granular media; because of cohesion, they exhibit a solid-like state even after reaching the incipient fluidization point. From this point to the peak, the excess pressure is accumulated in the form of internal stress. Interparticle attractive forces are crucial in this balance. They hinder the possibility that particles can detach from each other, slide each other, and ultimately use the drag force to untangle the internal friction. Then, while cohesion dominates, the internal structure buffers the excess pressure playing the role of a spring.

Eventually, when the gas flow rate is large enough to overcome the internal friction, the powder breaks and releases all the excess pressure accumulated. The result is a characteristic leap from which the pressure drop oscillates over the sectional weight of the powder bed, exhibiting a liquid-like state: the fluidization regime.

It is worth mentioning that, at the peak, the internal structure of the bed bears the maximum tensile strength.^31^ Experimentally, the fracture happened near the bottom of the bed, as expected theoretically.^35^

### 3.2 Packing Effect

Figure 5 displays the packing effect under standard laboratory conditions: 25 °C and 1 atm. As mentioned above, it compares the fluidization curves for raw samples subjected to different preconsolidations. The graph on the left shows the fluidization curves in samples with no additional consolidation. The consolidation stress due to gravity is indicated as σ_*c*_ = *W*, which represents the sectional weight of the powder bed. On the right side, the graph profiles those samples consolidated at 2 kPa.

Figure 5 reveals that preconsolidated samples exhibited larger values in tensile strength than those powders settled under the sole influence of gravity. Indeed, consolidation favors tighter packings that result in wider contact areas, which, in turn, boosts powder cohesion. As expected, this effect is sharper in 15 μm samples as cohesion increases with the surface-to-volume ratio. Thus, smaller particles are more vulnerable to stagnate each other as they come into contact while consolidating the sample. In fact, consolidation did not seriously affect the tensile strength in powders with little cohesion at room temperature (that is, 30, 45, and 60 μm series in Figure 5).

We shall return to this question after discussing the mechanical model (Sect. 4.2, Figure 13).

### 3.3 Temperature Effect

According to what has been mentioned before, preloads may change the internal friction of the bed. Consolidation increases cohesion by leading to tighter packings, which result in wider contact areas. Yet, contacts become wider too as particles soften at high temperatures.^1,14^

**Figure 6:**
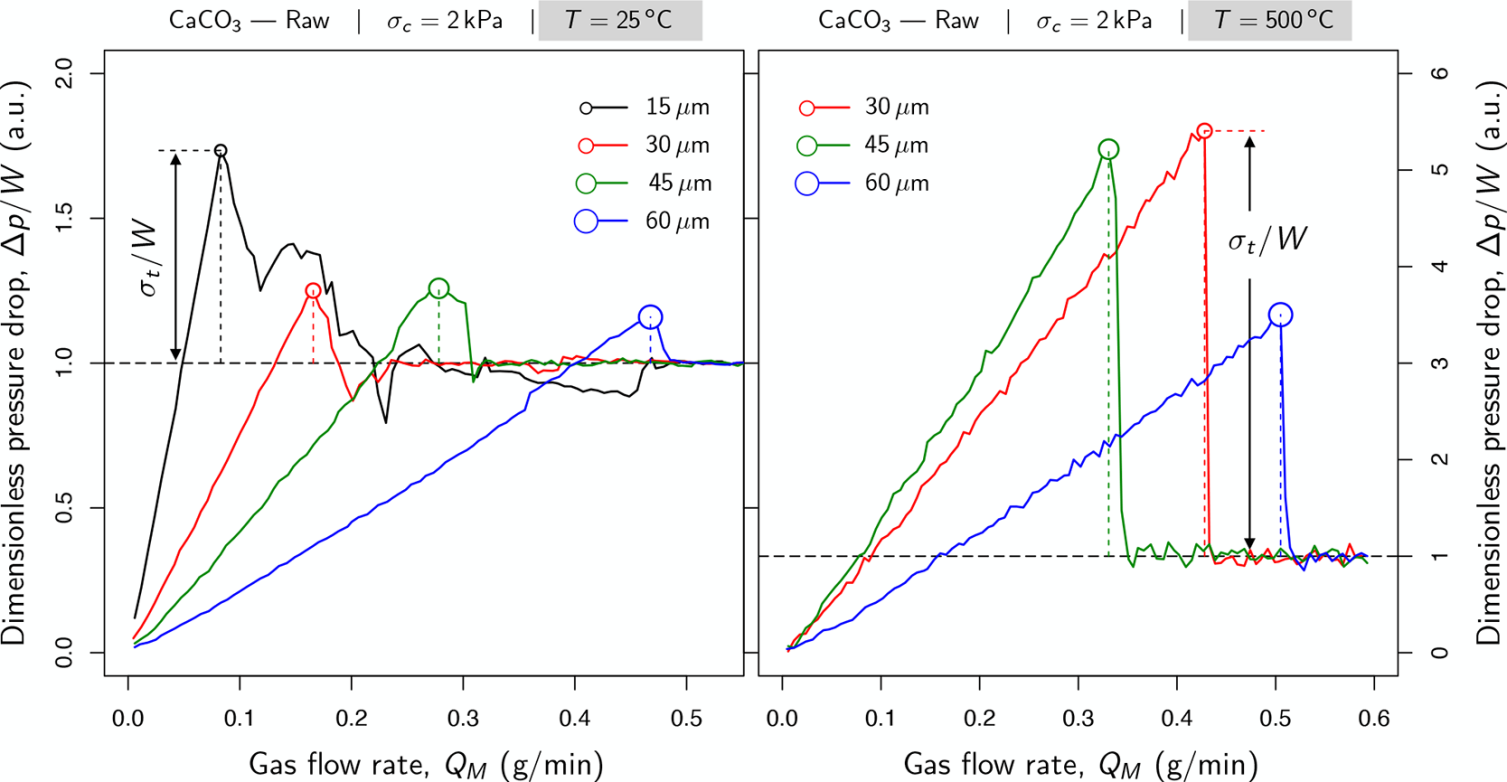
Effect of temperature (*T* = 500 °C). The gas pressure drop (Δ*p*) across the powder bed is plotted as a function of the mass flow rate; Δ*p*/*W* expresses the pressure drop in arbitrary units using the sectional weight of the powder bed (*W* ≈ 370 Pa).

Figure 6 details the effect of temperature in tensile strength. Graphs in Figure 6 contrast raw samples consolidated at 2 kPa: (left) at room temperature and (right) at 500 °C. As expected, the tensile strength escalated for temperatures close to the Tamman point in limestone (565 °C^36^). Interestingly, at 500 °C, samples with particle sizes about 15 μm displayed such a remarkable degree of cohesion that they could not be fluidized for the mass flow rates explored in this work. For this reason, the corresponding series (15 μm) was disregarded on the right graph of Figure 6.

### 3.4 Nanosilica Shielding Effect at High Temperatures

Previous Figures 5 and 6 highlight how the powder cohesion is modulated mainly by two factors: size and temperature.

Temperature increased tensile strength considerably, even in samples with negligible degree of cohesion at room temperature (e.g., 60 μm). As the temperature approaches the Tamman point particles soften, and powder tend to bridge. Therefore, Tamman is a crossover from which the medium mutates into a highly cohesive material. In nanosilica, such a turnaround occurs around 1000 °C, which roughly doubles its counterpart in carbonates, CaCO_3_. Not surprisingly, nanosilica coatings have shown to be an effective remedy to control the effect of temperature in cohesion,^1,30^ particularly in limestone powders with particles around 45 μm.

**Figure 7:**
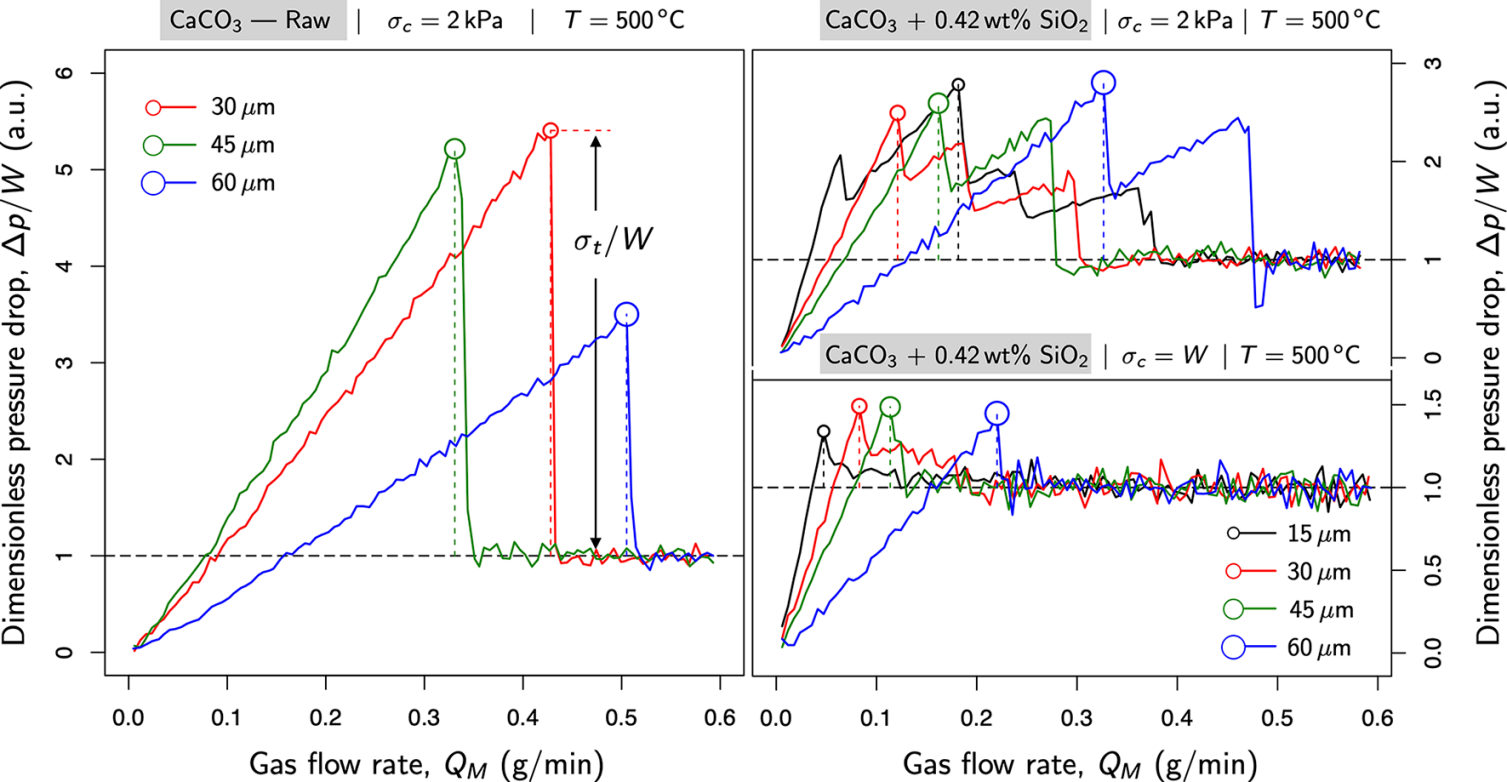
Nanosilica shielding effect at high temperature (*T* = 500 °C). The gas pressure drop (Δ*p*) across the powder bed is plotted as a function of the mass flow rate; Δ*p*/*W* expresses the pressure drop in arbitrary units using the sectional weight of the powder bed (*W* ≈ 370 Pa).

Figure 7 sketches the effect of nanosilica coatings at 500 °C for different particle sizes. On the left side, fluidization curves were plotted for raw samples consolidated at 2 kPa. On the right side, powders coated with nanosilica at 0.42 wt % and consolidated: (top) at 2 kPa and (bottom) under their own weight. Those samples covered with nanosilica reduced the tensile strength, even with a minimum amount of this material. The coverage helped to fluidize even the most cohesive samples (15 μm). More importantly, 15 μm powders coated with nanosilica exhibited lower cohesion than the raw samples with the largest particles (60 μm). Therefore, nanosilica coatings mitigate the rise in cohesion at high temperatures, even at temperatures close to the Tamman point.

**Figure 8:**
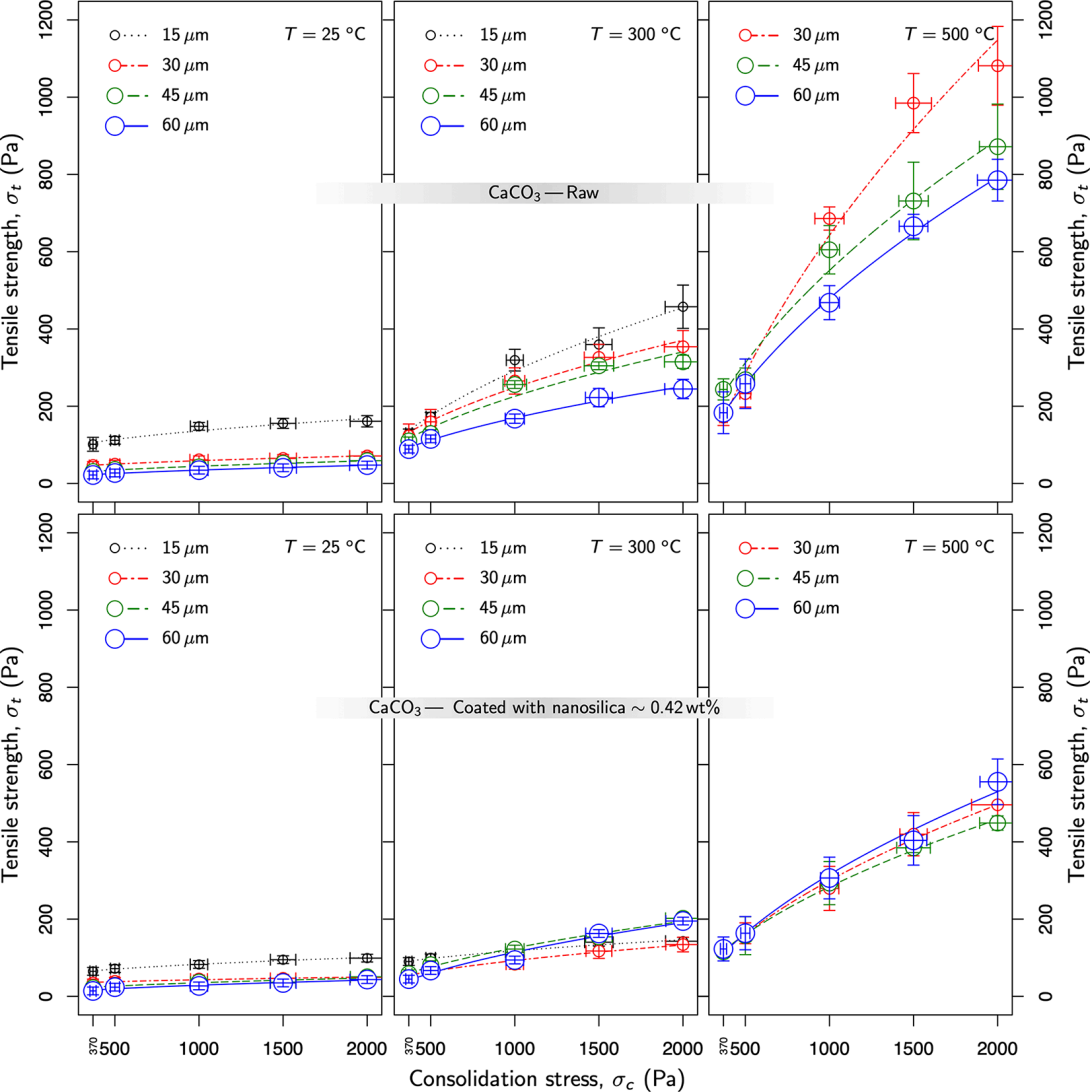
Tensile strength versus preconsolidation stress as a function of temperature. Lines map the sublinear trend reported in previous studies.^1,14^ Raw samples were represented at the top, whereas powders coated with nanosilica (at 0.42 wt %) were plotted at the bottom.

Figure 8 compiles the tensile strength for different samples subjected to preconsolidation stresses up to 2 kPa, and temperatures that varied from 30 to 500 °C. The top row alludes to raw samples, whereas the bottom one refers to samples coated with nanosilica at 0.42 wt %. The graphs show how the coverage of nanosilica depletes the promotion of cohesive forces at high temperatures. The tendency was even more acute for temperatures closer to the Tamman crossover. From 300 to 500 °C, cohesion was reinforced by a factor of 3 (average) in the raw samples. In contrast, powders coated with nanosilica at 0.42 wt % halved the increase observed in raw samples (bottom row in Figure 8).

Having shown the benefit of nanosilica coatings, would it be advantageous to add more nanosilica? The following paragraph tackles this question, paying special attention to larger particles.

### 3.5 Impact of Nanosilica Content on Powder Flowability

Here the analysis focused on the flowability of these powders. The flowability factor (*f f*) measures the ease with which powders can be handled while transporting the granular material from one sector to the other.^1,30,37,38^ This factor is reported in literature^37,38^ and referred to the unconfined yield strength for shear testers. Therefore, it is most often defined as the ratio between the consolidation stress used to preload the sample and the unconfined yield strength of the powder. However, this work monitored the tensile strength in powders subjected to uniaxial tensile stress. Nonetheless, previous studies^39,40^ showed that uniaxial and shear measurements should not differ significantly when performed in similar conditions. Correspondingly, an effective flow factor can be defined as the ratio between the preconsolidation stress applied to the sample and the tensile strength inferred from the peak of the fluidization curves:
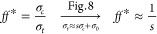
3where  represents the slope of the curves plotted in Figure 8, fitted by a linear model for the sake of simplicity.

Typically *f f* (and f f*) values greater than 10 are characteristic of powders with almost a negligible level of cohesion, which means those granular materials flow easily. The profiles in Figure 8 confirmed this trend. All the samples treated in this work exhibited a relatively flat slope (σ_*t*_/σ_*c*_) at room temperature. In these circumstances, eq 3 prescribes larger values in *f f*, which matches the observations with powders flowing easily. On the contrary, the profiles changed drastically at higher temperatures (Figure 8, right). As cohesive forces become dominant, flowability plummets. When *f f* exhibits values below 4, the granular dynamic is mainly governed by cohesion, and powders can hardly be fluidized. Eventually, if *f f* is below 2, powder behaves as a very cohesive granular medium with a quite limited possibility to fluidize.

**Figure 9:**
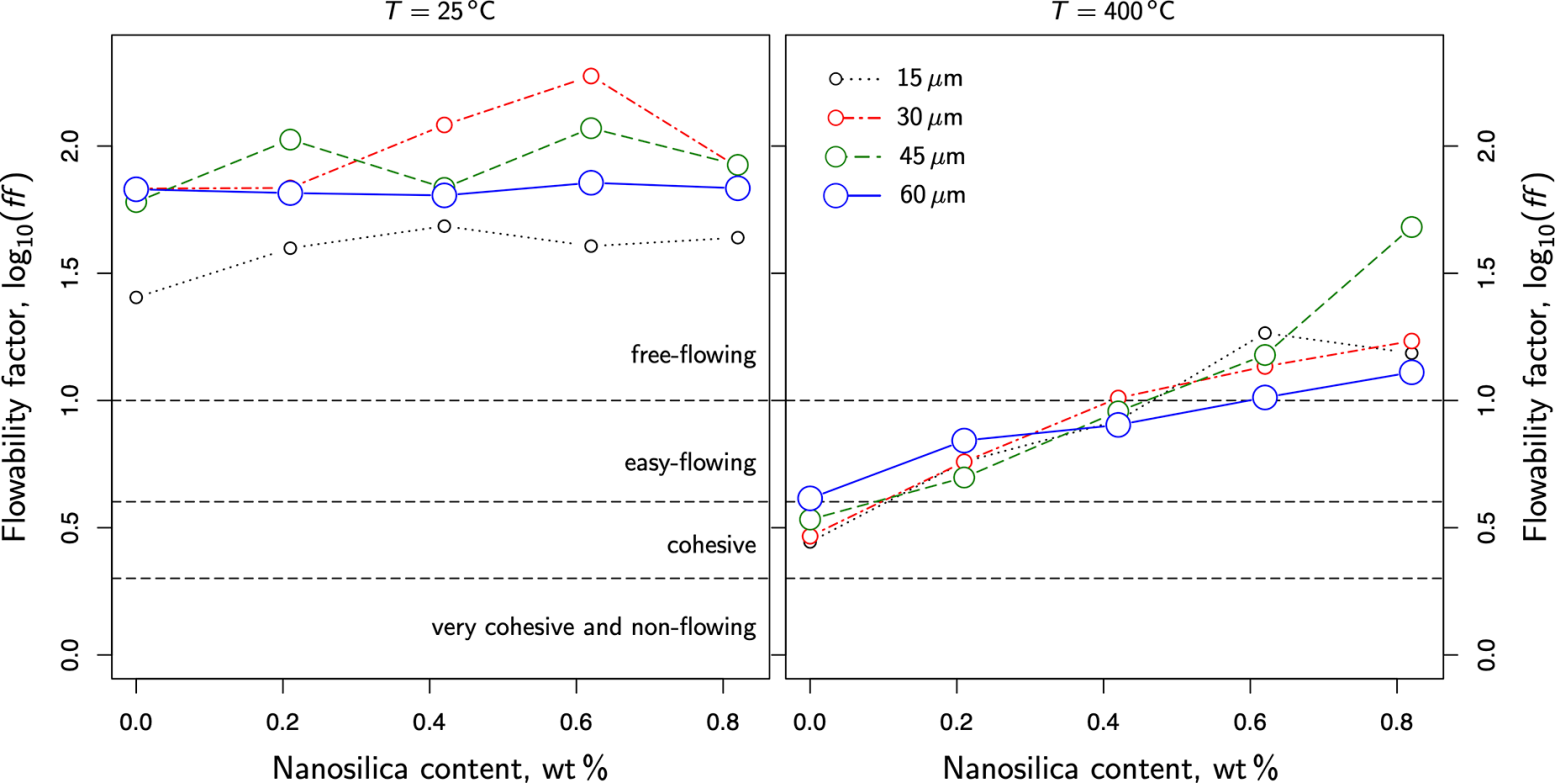
Effective flowability factor in logarithmic scale.

As flowability is critical in production environments, Figure 9 explores how nanosilica content modulates the granular flow regime in fine limestone powders. The left side of Figure 9 monitors the flowability of different samples at room temperature. As observed in previous graphs (Figure 5), all powders exhibited a negligible degree of cohesion at room temperature. In these circumstances, nanosilica coatings did not affect powder flowability significantly (Figure 9, left). However, the right side of Figure 9 unveils a more dramatic nanosilica effect at high temperatures. All the samples explored in this work, regardless of the size, lay within a free-flowing regime at high temperatures with just 0.82 wt % of nanosilica content. Without this additive, they were all in the cohesive region, where the powders could hardly be fluidized. Interestingly, from the point at which the samples reach the free-flowing regime (around 0.42 wt %), doubling the nanosilica content slightly improves the flowability of the samples. Furthermore, from then on, nanosilica has a minor impact in 60 μm samples.

**Figure 10:**
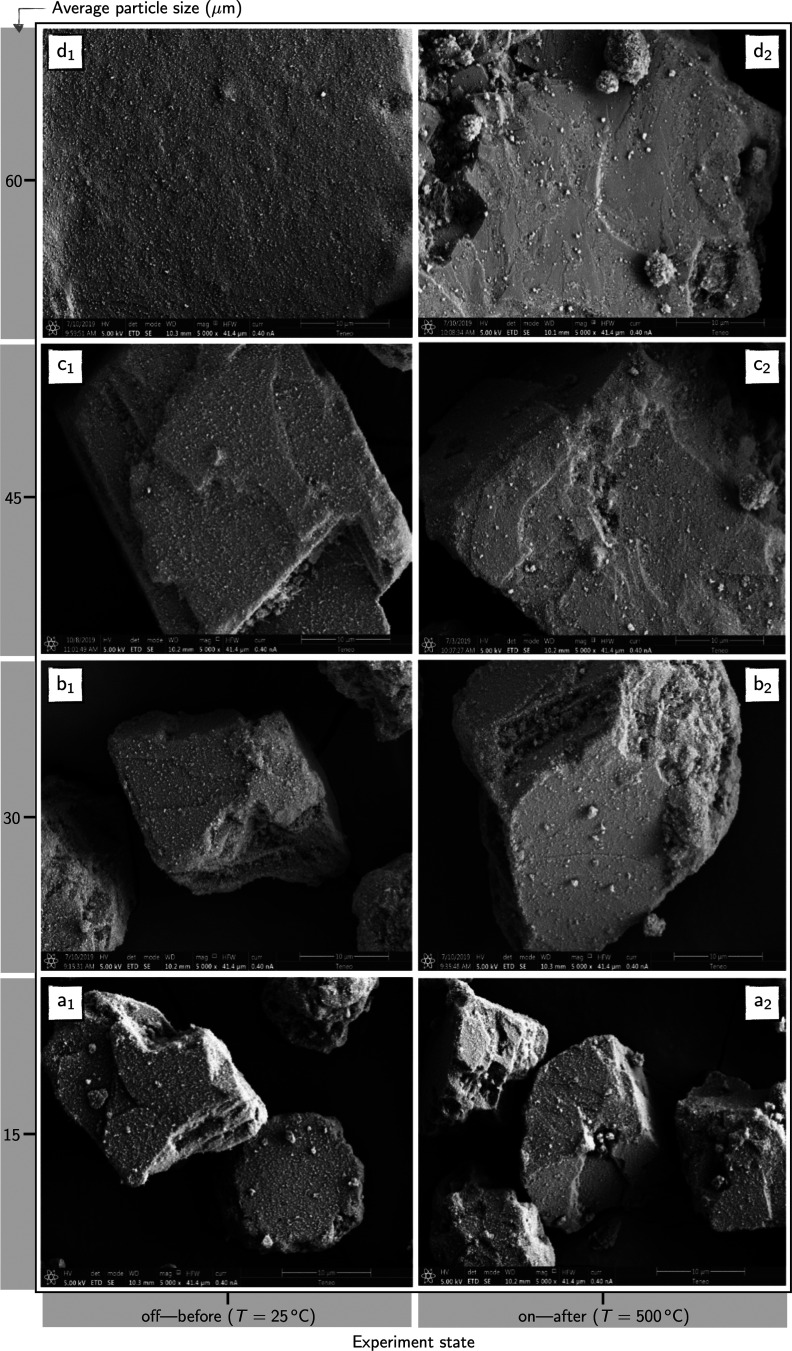
Scanning electron microscopy (SEM) images of limestone particles coated with nanosilica (0.42 wt %).

A close examination of the surface topology by scanning electron microscopy (SEM) images (Figure 10) revealed that only in 60 μm samples carbonate surfaces appear almost completely uncovered. All the samples exhibited a uniform coating before the experiment (Figure 10i_1_, *i* = {a,b,c,d} — left side). Moreover, Figure 10d_2_ shows the presence of large clusters of nanosilica, which suggests that nanosilica coatings are not stable at high temperatures in larger particles.

**Figure 11:**
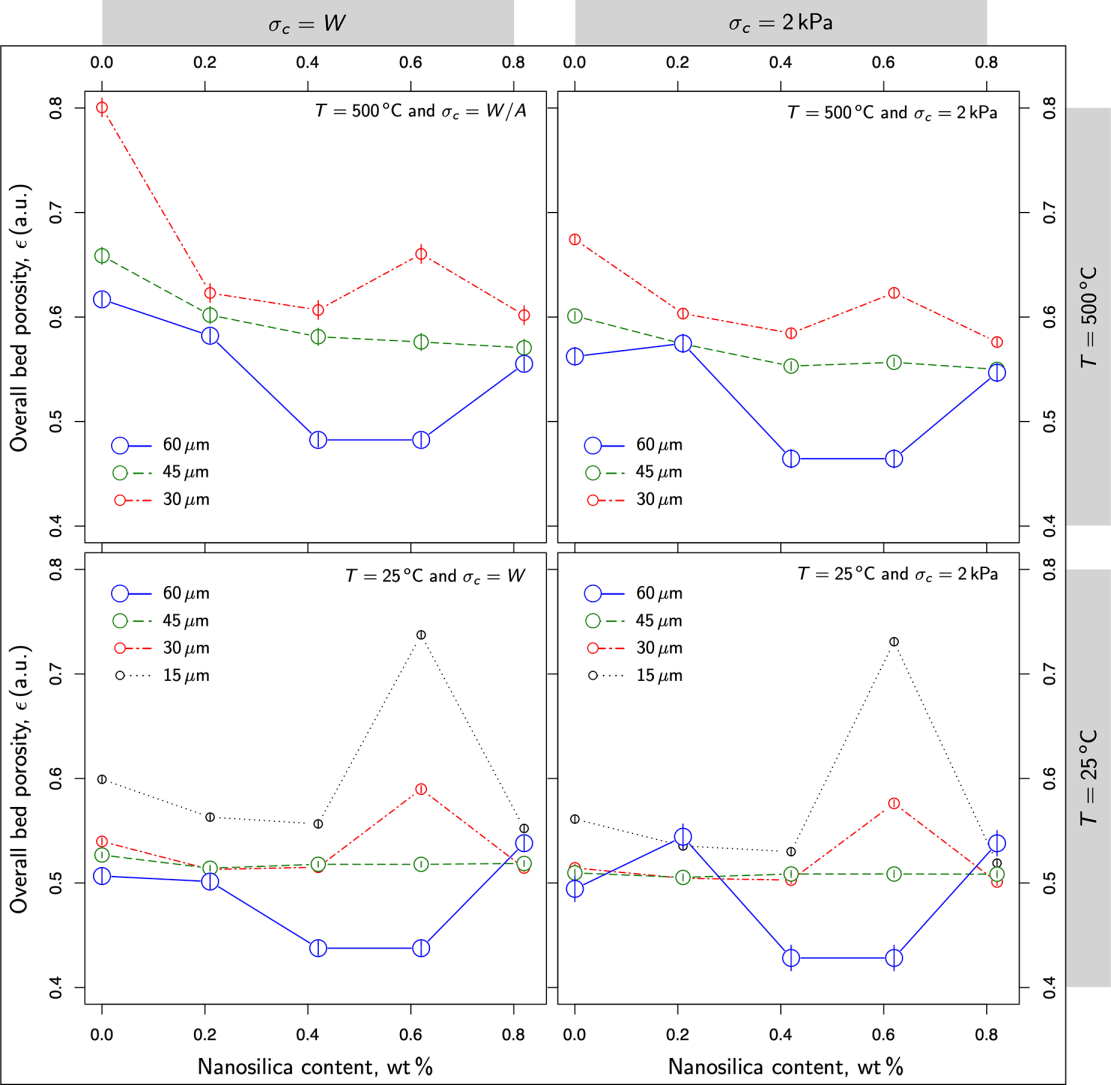
Overall bed porosity versus the nanosilica content added to the mixture.

Figure 11 sheds another view regarding the observations inferred from Figures 9 and 10. It shows the evolution of the bed porosity as the nanosilica content varied from 0 to 0.82 wt %. Armed with this complementary view, we tried to detail a qualitative reasoning for the stability of nanosilica coatings in larger particles. Theoretically, as the content of nanosilica increases, the layer grows faster on carbonate surfaces. Once carbonates are fully coated, the excess of nanosilica tends to fill the voids in the internal structure. As a result, the content of nanosilica alters the overall porosity either increasing or decreasing it. Below the surface saturation level, nanosilica layers the particles helping to mitigate the rise of cohesion at high temperatures. It provides a shielding effect that reduces the interaction between particles, allowing particles to easily roll and glide on each other. As the interaction between particles lessens, they rearrange in closer packings, and the bed porosity decreases accordingly. These qualitative predictions are in good agreement with the trends detailed in Figure 11.

But once the carbonates are fully coated, the excess of nanosilica tends to fill the voids, and porosity becomes increasingly determined by nanosilica. As it turns out, all the lines in Figure 11 converge as the nanosilica content increases. Because nanosilica particles are much smaller than carbonates, the saturation should match the minimum in the overall bed porosity.

According to this reasoning, only 60 μm samples reached the saturation from 0.42 wt %. In effect, larger particles exhibit larger surface-to-volume ratios. As a result, for the same weight ratio between nanosilica and carbonate, powders with larger particles have more nanosilica available to layer the surfaces. For this reason, samples with larger particles result in larger *surface active coverage* (SAC):^41,42^
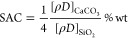
4where the subscripts CaCO_3_ and SiO_2_ allude to the carbonate and nanosilica particles, respectively. The parameters *D* and ρ in eq 4 refer to densities and particle sizes. According to eq 4, 60 μm samples were expected to reach the nanosilica saturation first.

## 4 Discussion

Interparticle contacts modulate the microscopic forces that ultimately govern powder cohesion. Cohesion depends largely on the ease with which the surface material deforms. Overall, particles tend to soften when the powder bed is consolidated (σ_*c*_) at high temperatures. At the contact, the macroscopic stress (σ_*c*_) becomes a local force, which is called the *pull-on force*, *F*_*c*_. In contrast, if an uplift force is applied to the bed, the granular medium undergoes a tensile stress that tends to break up the internal friction. These are the two types of stresses we controlled and monitored during the experiments. First, a downward gas stream was imposed over the bed to consolidate the samples. This stage promotes a more severe internal friction as consolidation becomes more important for higher flow rates. Later, an upward flow produces a drag force that applies tension to the powder bed as the gas flow rate exceeds the incipient fluidization. As a result, the drag force unloads the interparticle contacts progressively. When the powder bed fractures, the tensile stress peaks, and the local force at the contact reaches its maximum, which is called the *pull-off force*, *F*_*t*_. At this point, pull-off forces are strong enough to untangle the internal friction, and eventually, the bed crumbles and expands.

The next paragraphs detail how powder cohesion is related to particle plasticity and, more importantly, its role in the ability of powders to flow.

### 4.1 Flowability and Powder Cohesion — The Role of Attractive Forces

Powder cohesion is mainly governed by the microscopic forces that arise at interparticle contacts. It turns out that contacts are mediated primarily by a couple of factors: the stiffness (*E*) and the hardness (*H*) of the particles. These two factors are critical when the powder is loaded and exposed to high temperatures. In such conditions, particles soften, contact areas increase, and cohesive forces skyrocket.

This subsection concentrates on those experiments in which powder cohesion is governed just by the attractive contribution of the interparticle forces. Thus, it assumes nondeformable surfaces, focusing on the nature of the interaction between particles. Although rigid bodies represent an oversimplification in many cases, this approximation serves to dissect how the nature of different forces intervene in cohesion. The following subsection extends this approach, analyzing the role of particle plasticity and its relationship with temperature and powder cohesion. Therefore, here, we center on those experiments at room temperature, in which samples were not consolidated—more precisely, those in which samples were consolidated under their own weight. In these circumstances, the granular Bond number makes it possible to characterize powder cohesion based solely on a static picture of attractive forces:^43,44^
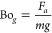
5where *F*_*a*_ represents the minimum *pull-off* force needed to separate two particles at contact.

According to eq 5, Bo_*g*_ ≫ 1 means that cohesive forces dominate—cohesive regime. As attractive forces control the powder dynamic, each contact through the internal structure contributes to bridging the powder. As a result, Bo_*g*_ ≫ 1 reflects powders that hardly fluidize. On the contrary, Bo_*g*_ ≪ 1 highlights that inertial forces govern the internal friction—inertial regime. In this latter case, the capability to fluidize the bed is limited mostly by the packing of the bed.

Certainly, there is a plethora of forces lurking under the attractive behavior between particles. Among all of them, the most important ones in fine powders are usually capillary, magnetic, electrostatic, and van der Waals forces.^44−47^ Capillary forces, for example, can be neglected^48^ because the setup was equipped with filters and dryers that run the experiments with dry samples and maintain the circuit free from pollutants and humidity. Similarly, electrostatic forces do not play a significant role since van der Waals forces dominate over electrostatic ones in uncharged fine powders.^40,49,50^ Regarding magnetic forces, limestone samples exhibited almost no magnetic behavior. Eventually, retardation effects can be neglected as well.^51^ This is because the contact in real surfaces is most often shaped by the ineradicable asperities of the surfaces. The size of these irregularities is, typically, of the order of 0.1 μm for particles around 10 μm.^52,53^ Furthermore, 1000 Å, or equivalently 0.1 μm, is precisely the distance from which the retardation effect starts to weaken the London interaction.^51^ More precisely, the force approximately halves at 0.1 μm. For this reason, the following analysis neglects the role of propagation that accounts for retardation effects, considering that there is no significant change in the order of magnitude for the average size of the asperities. In these circumstances, van der Waals forces mediate the interaction between carbonates. But before going any further, it is worth mentioning why a molecular approach is suitable to model the interaction between particles. As extended bodies, particles do not often exhibit a permanent dipole moment. When this is the case, as it is for CaCO_3_, particles represent thermodynamic systems in which the interaction between polar components cancels each other. However, this balance in particle volumes cannot be applied at the interfaces, where the particle surroundings change and forces unbalance accordingly. Because of that, the source of attraction between particles can be described by the interaction of molecules that occurs at the contact and its more immediate surrounding.^54^ All these conditions redound to the suitability of the molecular approach proposed by Hamaker to compute the attraction between two particles.^44,54,55^ Here is the expression for a pair of interacting spheres:
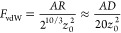
6where *A* is the Hamaker constant, typically around 10^–19^ J for most solids in vacuum;^54^*z*_0_ represents the equilibrium distance between the surfaces where attractive and repulsive contributions balance each other, typically ranging from 3 to 4 Å;^40,49,56,57^ and *D* = 2*R*, with *R* representing the reduced radius of the spheres: .

However, the cohesion prescribed by eq 6 is rarely observed experimentally.^58^ As mentioned above, the interaction is most often mediated by protuberances on the surfaces. The height of these irregularities is usually larger than the distance that typically limits the range of cohesive forces.^58^ Moreover, Rumpf^59^ and Rabinovich et al.^60,61^ showed that pull-off forces decrease significantly due to the roughness of the surfaces, even if the irregularities were very small in amplitude. For this reason, the reduced radius in eq 6 considers the contact between asperities, which results in *R* ≈ *R*_asp_ ≈ 0.1 μm.

In these circumstances, the granular Bond number may be evaluated as follows:^44^
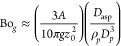
7where *D*_asp_ accounts for the size of the asperities, whereas *D*_*p*_ and ρ_*p*_ represent the particle size and density, respectively. The first parentheses in eq 7 reflects a factor with very little variation between particles of different species. In contrast, the second one points out a more determinant factor related to particle size and surface topology. Not surprisingly, eq 7 predicts that larger particle sizes lead to lower degrees of cohesion. This theoretical prediction agrees with the experimental results displayed in Figure 5 (graph on the left side). Therefore, when comparing the effect of different particle sizes, the intensity of cohesive forces diminishes quickly for larger particles:
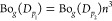
8where . To estimate the Bond numbers associated with the samples: (1) *A* ≈ 1.01 · 10^–19^ J for CaCO_3_, (2) *z*_0_ ≈ 4 Å, and (3) the radius of curvatures for asperities can be considered about 0.1 μm (as mentioned above). In particular, for samples of 60 μm, the granular Bond number turns out to be 2, approximately. Conversely, this value increases 2 orders of magnitude for particles about 15 μm (the smallest particles used in this work). The transition between the inertial and cohesive regimes is usually assumed to occur at Bond numbers around 1. It would be expected, then, to observe this transition from inertial to cohesive as the particle size decreases. This transition is clearly highlighted in Figure 5 (graph on the left side), where the data support the theoretical predictions about the evolution with the particle size.

**Figure 12:**
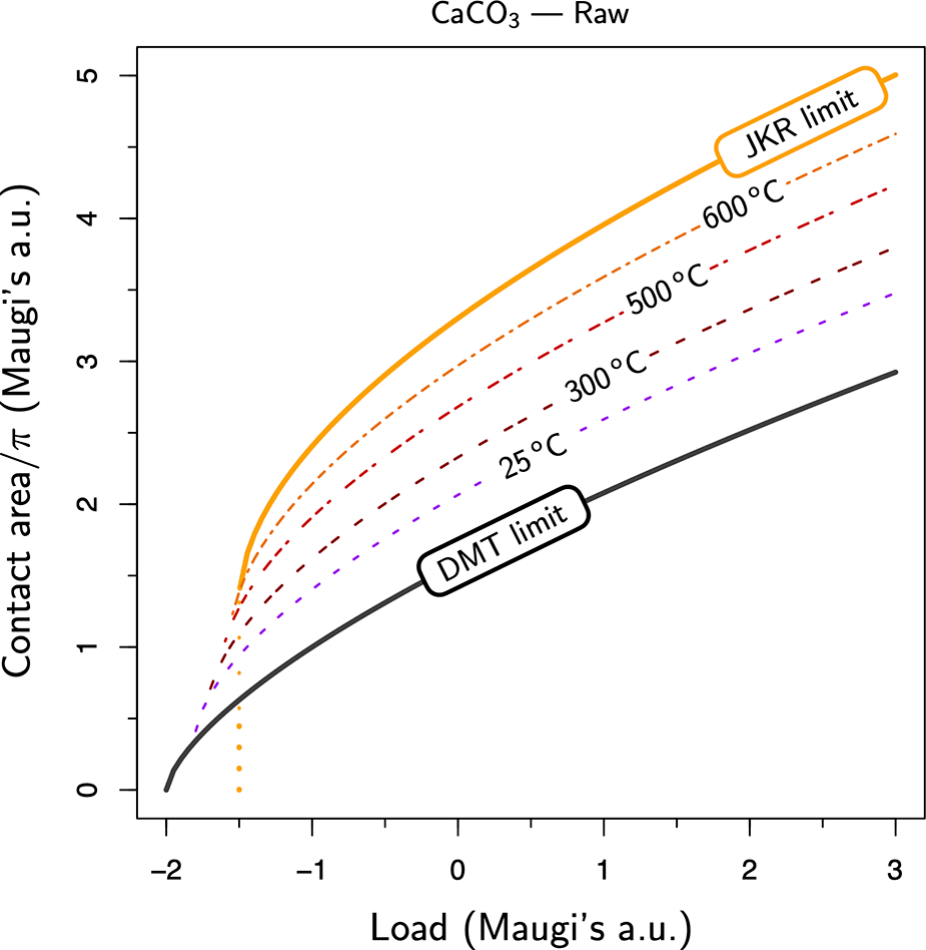
DMT–JKR transition as temperature increases. Contact area and load are expressed in Maugis’ units.^63^ Dashed lines trace the evolution of mechanical properties for limestone.^71^

However, those granular Bond numbers do not provide any glimpse about how the contact unfolds. It assumes tacitly that particles just undergo an attractive interaction as they approach each other. This image of the contact matches when particles are stiff enough so that the surface deformation is negligible while particles interact. Thus, this approach is based on rigid spheres, where collisions occur within very confined regions that tend to concentrate the load at a single point. Furthermore, if the contact happens between particles with small curvature radii, the repulsive forces in the van der Waals relation will be barely weighted. This is because the increase in curvature narrows the region where repulsive forces dominate. In summary, assuming rigid spheres and contacts controlled by asperities with small curvature radii, long-range cohesive forces govern the friction and powder flowability. In fact, drawing attention just to cohesive forces, the theoretical background aligns with the Bradley limit predicted by the DMT theory.^62^ Finally, the formulation plotted in Figure 12 tracks the suitability of these assumptions as the temperature approaches the Tamman point. This graph shows how the contact area varies with the local load; both magnitudes are expressed in Maugis units.^63^ The dashed lines in this figure illustrate the transition between DMT^62^ and JKR^64^ contacts according to the Maugis–Dugdale model.^63^ Each of those lines considers the variation in the mechanical properties in the CaCO_3_ samples at the corresponding temperature.^65^

### 4.2 Flowability and Powder Cohesion — The Role of Particle Plasticity

While the DMT approach is suitable for stiff and almost rigid spheres, the JKR model applies to softer surfaces. Figure 12 delineates the isotherms within these two limits. As the temperature varies from 25 to 600 °C, the isotherms shift progressively from the DMT limit to the JKR counterpart. It is foreseeable then that CaCO_3_ surfaces soften at high temperatures as they contact each other. Softer surfaces, in turn, result in wider contact regions that increase the powder cohesion. It is clear in Figure 12 that from 300 °C forward, the rigid-spheres predictions of the DMT models cannot be applied to explain the rise in tensile strength. At room temperature, though, isotherms suit relatively well with the DMT approach.

**Figure 13:**
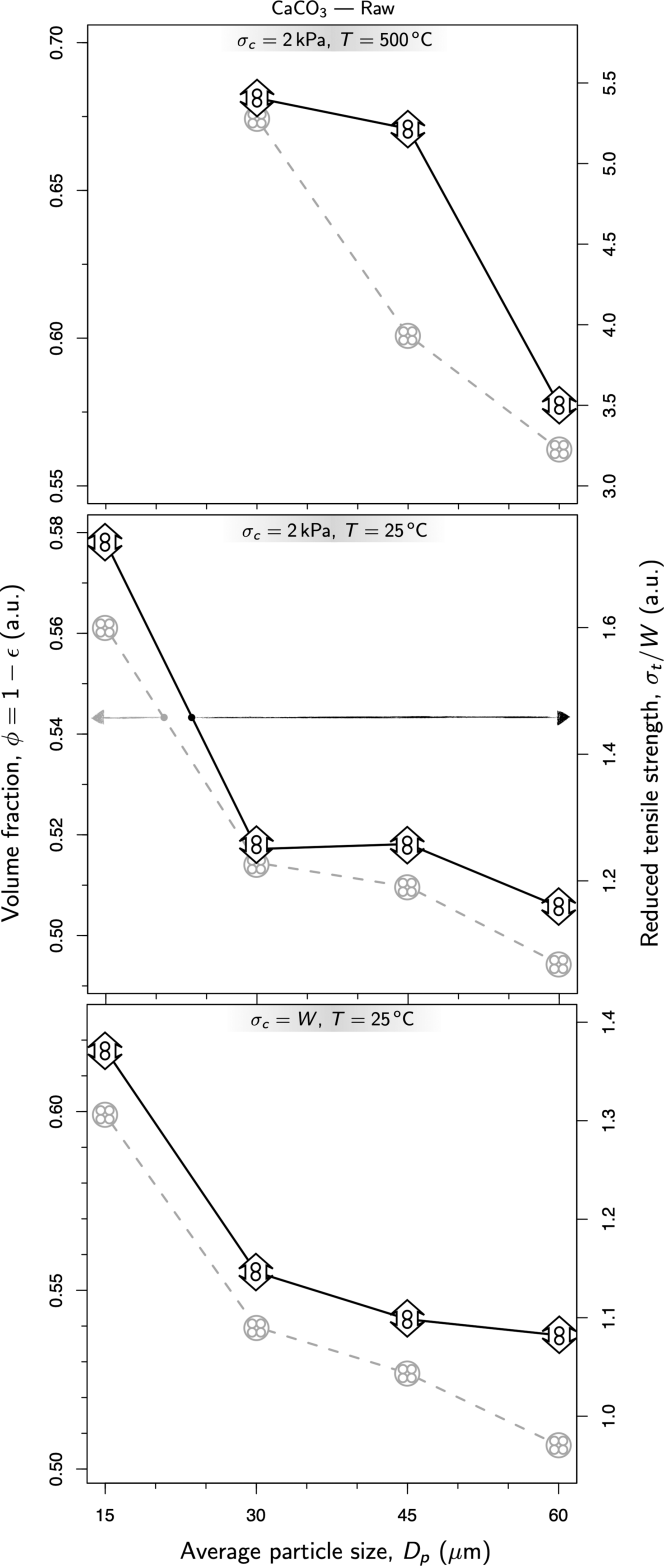
Packing effect for different samples explored in this work. Primary *y*-axis refers to the volume fraction, whereas the secondary one (at the right side) alludes to the reduced tensile strength, σ_*t*_ (expressed in terms of the sectional powder weight, *W* ≈ 370 Pa).

However, if the softening effect is not significant at room temperature, another question arises: What is the mechanism that induces more cohesive dynamics with larger consolidations? This issue was tackled in the Results section (Sect. 3), outlining the role of packing. Here, Figure 13 shows how the volume fraction parallels the trend followed by the tensile strength. Volume fractions are logged on the primary *y*-axis on the left side; a gray dashed line is used to represent this magnitude. On the other hand, the values of tensile strength are monitored on the secondary *y*-axis on the right side. In this case, a solid black line was used to plot the trends. The bottom graph describes the evolution of the volume fraction with the particle size. In this case, samples were consolidated under their own weight at room temperature. The tendency of the volume fraction matches the evolution of the tensile strength with the particle size. This parallelism suggests that the packing is the primary mechanism behind the changes in the powder cohesion at room temperature (far enough from the Tamman temperature). If so, the use of a downward flow to consolidate the samples should promote better packings. Moreover, tighter structures would result in wider contact areas, which eventually leads to more cohesive structures. This theoretical prediction agrees with the data in the middle graph, where the samples were subjected to a consolidation stress of approximately 2 kPa at room temperature. However, the match between volume fraction and tensile strength is not so clear when the temperature approaches the Tamman point (top graph). At 500 °C, in 45 μm samples consolidated at 2 kPa, tensile strength and volume fraction exhibited quite different evolution with a significant gap between the relative change of these magnitudes. Recalling Figure 12, contacts at 500 °C are modulated by softer surfaces, which boost cohesive forces as the contact region widens significantly. Then, the tensile strength is expected to blow up from this point forward. Powder beds with an average particle size of about 30 μm exhibited less compact packings than those observed in the middle graph while transitioning from 45 to 30 μm samples at room temperature. This is the hallmark of cohesive forces, which typically lead to loose packings as cohesion is hindered so that particles can shear off to sideward positions. The effect was so noticeable in powders with an average particle size of about 15 μm that these samples could not be fluidized for the flow rates used in this work.

Flowability combines the action of cohesive forces, stiffness, and particle plasticity.^12^ As mentioned before, the granular Bond number captures just the ratio between the attractive forces and the particle weight. Thus, it ignores how changes in the surface profile can modulate the force needed to separate the particles (*pull-off* force). Far from the Tamman point, when particles behave as rigid bodies, the Bond number is an excellent predictor to control the powder flowability. However, as temperature goes closer to the Tamman crossover, the surface deformation modulates the contact dynamic. In these circumstances, because contacts involve elastic or plastic deformations more sophisticated models are required to describe the variation in the powder flowability.

Indeed, the molecular perspective anticipates the need to consider the surface deformation to map the flowability regimes at high temperatures. Here, a key question is how a molecular point of view can be tied to macroscopic measurements. In this regard Rumpf’s formula^66^ connects macroscopic stresses (σ_*i*_, either tensile strength or consolidation stress) and local forces (*F*_*i*_, either pull-off or pull-on forces):
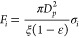
9where ξ = ξ(ϵ) is the coordination number defined as the number of contacts per particle. A simple estimation of the coordination number can be calculated using the formula:^67,68^
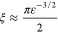
10

According to Figure 11 the overall bed porosity lies within the range from 0.4 to 0.6, which means coordination numbers from 4 to 6.

However, eq 9 is based on simple geometrical arguments assuming the contact occurs at a single point. To consider the case that particles can indent each other beyond the elastic limit, some adjustments are in order in eq 9, especially to think about the possibility that one or both particles suffer a permanent deformation:^44,69,70^
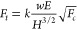
11where *k* is a factor that accommodates the peculiarities of both the model and deformation assumed during the load process; *w* is the work of adhesion, *w* = 2γ, with γ representing the surface energy; and *E* refers to the reduced Young modulus:
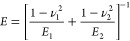
12where ν_*i*_ and *E*_*i*_ are the Poisson ratio and Young’s modulus of the two solids (*i* = 1, 2) in contact, respectively.

Although contacts rarely happen on a single load point, as prescribed by eq 11, this relation foresees a sublinear trend that matches the observation in Figure 8.

Indeed, eq 11 sheds light on the role played by the material properties in pull-off forces. It is not surprising, then, that, in limestone, tensile strength skyrockets as properties such as elastic modulus, hardness, and Poisson ratio plummet when the temperature approaches the Tamman crossover.^65^ Within this context, nanosilica coatings can mitigate (theoretically) the rise in cohesion because they make contacts more resilient to high temperatures. In fact, nanosilica exhibits a Tamman temperature that roughly doubles the limestone value, which ensures less deformable contacts. Therefore, in fully coated particles of CaCO_3_, nanosilica mediates the contact, ensuring lower cohesion and better flowability factors. This theoretical expectation fits perfectly with the observations registered in Figures 8 and 9.

However, the resilience of these coatings suffers in those cases with larger particle sizes, particularly in 60 μm samples (Figure 10). Three factors may affect the resilience of these coatings directly, making samples with larger particles especially vulnerable to erosion in nanosilica layers:

(i) Surface active coverage (SAC). For a given amount of material and nanosilica, samples with larger particles have more nanosilica content available to layer the powders. Consequently, larger particles expose smaller effective areas. At the same time, once the surface is entirely layered, the nanosilica excess drifts with the gas stream, tending to agglomerate. Therefore, if agglomeration happens, it will occur first in samples with larger particles.
(ii) Force nature that binds the coating. There is no evidence to specify the nature of the forces that stick nanosilica particles to carbonates. Electrostatic interaction is hardly the force that binds the nanosilica coverage to carbonate surfaces. Any charge excess would be equalized over time because of the continuous contact between particles. Indeed, van der Waals and electrical double layer forces contribute more significantly to cohesion. These two forces usually induce electrical pressures of the same order of magnitudes. However, van der Waals forces decrease with 1/*D*_*p*_^3^, whereas double-layer forces remain almost constant, even through macroscopic distances. If van der Waals forces were the force that binds the nanosilica coatings to the carbonate surfaces, it would be easier to detach the coverage in those samples with larger particle sizes.
(iii) Packings. Packing is another factor that might contribute to the erosion of the nanosilica layers observed in 60 μm samples. As discussed previously, powder beds with larger particle sizes exhibit a lower degree of cohesion, which leads to closer packings. Tighter internal structures shape narrower channels, making the internal cavities more vulnerable to the erosion caused by large aggregates.

## 5 Conclusions

Particle size plays an important role in the efficiency of nanosilica coatings in limestone powders. This is a critical issue for thermochemical storage systems based on the calcium looping process. Today nanosilica is used in these facilities to ease the handling of cohesive granular media, such as limestone powders. Indeed, experiments demonstrated that nanosilica coatings control the upsurge in cohesion at high temperatures, even in small amounts. However, the results also showed that the efficiency of these coatings decreased significantly for particle sizes larger than 60 μm. In light of these findings, we believe that the analysis and discussion presented in this work contribute to the still open debate about the optimal particle size in fluidized bed and entrained flow reactors.

The mechanical analysis revealed that these layers provide a higher degree of hardness and thermal resistance than raw carbonates. Classical theoretical models such as DMT and JKR were used to frame the analysis, determining the role of particle plasticity in the rise in cohesion at high temperatures. While the DMT limit describes the contact behavior quite well at low temperatures, the JKR limit suits the powder behavior observed at high temperatures (close to the Tamman point, when particles soften). The good agreement between these theoretical models and the experimental data indicates that contact mutates from rigid to elastoplastic as the temperature approaches the Tamman point. Thus, deformable contacts are the primary cause of the significant rise in cohesion at high temperatures.

According to the mechanical hypothesis, the use of nanosilica provided an additional thermal resistance to surface deformation at high temperatures. The hardness in nanosilica is similar to that of carbonates, but the Tamman point roughly doubles its carbonate counterpart. Interestingly, when carbonates were entirely coated with nanosilica, the tensile strength roughly halved compared to raw carbonates at 500 °C. Therefore, the experimental data confirm that nanosilica makes carbonates more resilient to the rise in cohesion at high temperatures.

However, nanosilica coatings showed little resilience in samples with larger particles (60 μm). In this regard, SEM images confirmed that the surface of 60 μm particles was barely coated with nanosilica. More importantly, unlike those samples with smaller particles (below 60 μm), doubling the content of nanosilica improved the flowability, but just slightly. These two striking features highlight a crossover around 50 μm, from which nanosilica layers are no longer stables. Examining these two peculiar traits, we suggest that the binding force could be related to the van der Waals contribution instead of double-layer forces, among others. Although there is no conclusive evidence to reveal the nature of the force that sticks these layers to carbonates, our discussion indicates a route to explore more resilient and effective nanosilica coatings. Indeed, this is a critical point for the future of thermochemical storage units since it would propel the design of more efficient treatments.

In summary, nanosilica coatings exhibit a turning point for particles around 50 μm. The efficiency of these layers declines for larger particles (above 50 μm) that still increase cohesion significantly at high temperatures. This limitation applies especially to applications based on fluidized and entrained flow reactors. Today, powder technologies based on these reactors still demand smaller particle sizes to improve the reactivity of granular media. But dealing with smaller particles requires more efficient treatments to control the rise of cohesion, especially at high temperatures. For these reasons, these findings could also be of interest to other engineering niches that tackle similar scenarios, such as flour or cement industries, to name a few.
